# Genome-scale DNA sequence data and the evolutionary history of placental mammals

**DOI:** 10.1016/j.dib.2018.04.094

**Published:** 2018-05-01

**Authors:** Shaoyuan Wu, Scott Edwards, Liang Liu

**Affiliations:** a Jiangsu Key Laboratory of Phylogenomics & Comparative Genomics, School of Life Sciences, Jiangsu Normal University, Xuzhou, Jiangsu 221116, China; b Department of Organismic & Evolutionary Biology, Museum of Comparative Zoology, Harvard University, Cambridge, MA 02138, USA; c Department of Statistics and Institute of Bioinformatics, University of Georgia, 310 Herty Drive, Athens, GA 30606, USA

**Keywords:** Phylogenomics, Mammal, Alignment

## Abstract

We present a genomic data set comprised of the coding DNA sequences of 5162 loci from 90 vertebrate species, including 82 mammals. The loci were aligned with their protein sequences. The aligned protein sequences were then back translated into their original DNA sequences. The alignments were further filtered to remove individual sequences from each alignment exhibiting long branches or other unusual features. The data is deposited in figshare (http://figshare.com/articles/cds_5162.zip/6031190) and will be useful as a test data set for large-scale phylogenomic analysis.

**Specifications table**

**Table:** 

Subject area	*Biology*
More specific subject area	*Molecular Evolution*
Type of data	*text file*
How data was acquired	*Sequencing and Blasting in GenBank*
Data format	*Filtered*
Experimental factors	*The alignments were trimmed and filtered*
Experimental features	*Phylogenetic analysis of the alignments*
Data source location	*GenBank*
Data accessibility	*Dryad*

**Value of the data**

• The data reveal the evolutionary relationships of placental mammals.
• The data, combined with fossil information, can be used to estimate the divergence times of placental mammals.
• The data can be used to evaluate the performance of various phylogenetic methods in estimating phylogenies and divergence times.

## 1 Data

The data consist of DNA sequence alignments derived from Liu et al. [1], representing the CDS sequences of 5162 loci from across 90 vertebrate species, including 82 mammals. Liu et al.'s [1] original data were unaltered natural sequence alignments, which are here processed further to remove potential low-quality regions and indels. Whereas a critique of the original data set [2] was unnecessarily pessimistic about the consequences of any alignment errors, we recognize that, although our original alignments correctly designated each codon position in our analyses, the alignments themselves were not guided by amino acid codons and therefore could be improved. Here we present such improved alignments, which should be valuable as a test case in phylogenomic analysis.

## 2 Experimental design, materials and methods

Liu et al. [1] performed a DNA-based alignment. In contrast, we aligned the 5162 loci based on the protein sequences using the program Mafft v6 [3], where the protein sequences were translated from their original DNA sequences using the program EMBOSS [4]. The aligned protein sequences were back translated into DNA sequences according to their original DNA codon usage. We then employed the program trimAl [5] with the option -*gappyout* to trim the aligned DNA sequences. Liu et al. [1] had identified loci that may be misaligned by calculating the ratios between the maximal and average branch lengths of each of the gene trees and then removing entire alignments from the dataset if they did not pass the long-branch gene tree test. Here we instead applied Philippe et al.'s [6], [7] protocol to identify individual sequences within each locus that might generate long-branches due to misalignments or misassemblies. We then removed the individual sequences flagged by this protocol, rather than removing entire genes from the dataset. In the alignment screening protocol, a reference tree was reconstructed from the concatenated sequences of 100 genes. For each gene, the reference tree was pruned to match the taxa of the gene, and the branch lengths of the pruned reference tree were estimated from the gene alignment. If an estimated branch length of this new tree was more than 5 times the original branch length of the pruned reference tree, the sequence corresponding to this branch was removed from the gene alignment. However, after performing this protocol, we still observed some long branches remaining in maximum likelihood (ML) gene trees after pruning. We therefore further filtered the alignments by comparing ML gene trees with the pruned reference tree. In this last step, for each gene, if the length of a terminal branch of the ML gene tree was more than 5 times the length of the corresponding terminal branch in the pruned reference tree, the corresponding sequence was removed.

**Fig. 1:**
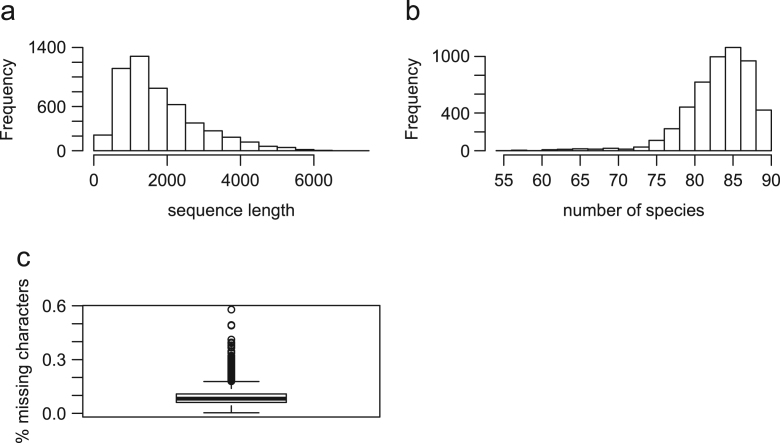
The summary of the alignments for 5162 loci. (a) The histogram of the sequence length across 5162 loci. (b) The histogram of the number of species across 5162 loci. (c) The boxplot for the proportion of missing characters across 5162 loci. Missing characters include gaps and ambiguous characters.

The filtered alignments of 5162 loci from 90 species are summarized with respect to the alignment length, the number of missing sequences, and the proportion of missing characters (Fig. 1). The length of each alignment, including gaps, ranges from 204 bp to 7017 bp with the average length being 1773 bp (Fig. 1a). The total length of the concatenated alignment is 9,150,597 bp. On average, there are 6 missing sequences per gene, whereas 86% of 5162 genes contain more than 80 species (Fig. 1b). The average proportion of missing characters across loci is 9% (Fig. 1c). These statistics compare well to our original data set, which was only 4388 alignments comprising 13,040,111 bp. These improved alignments are deposited in figshare (https://figshare.com/articles/cds_5162_zip/6031190) for further analyses. We have already demonstrated [8] that re-analysis of a 60-gene subset of this improved data set does not change the results presented in Liu et al. [1], and we plan to present a more comprehensive re-analysis of this data set elsewhere. We hope this data set is used widely as a test case for phylogenomic analysis and dating of divergence times.
